# Association between breastfeeding and lower risk of behavioral problems in children: a systematic review and meta-analysis

**DOI:** 10.3389/fped.2026.1867649

**Published:** 2026-06-12

**Authors:** Xue Liu, Miao Liu, Hongli Chen, Yinan Zheng, Qian Zhao, Zhongli Li, Yuling Zhou, Lilian Chen

**Affiliations:** 1 College of Nursing, Shanxi University of Chinese Medicine, Jinzhong, Shanxi, China; 2 Department of Child Health Care, Shenzhen Guangming Maternity & Child Healthcare Hospital, Shenzhen, Guangdong, China; 3 Department of Child Psychology Rehabilitation, Shenzhen Maternity and Child Healthcare Hospital, Southern Medical University, Shenzhen, Guangdong, China

**Keywords:** behavioral problems, breastfeeding, children, externalizing behavioral problems, internalizing behavioral problems, systematic review, meta-analysis

## Abstract

**Objective:**

To systematically evaluate the association between breastfeeding and the risk of behavioral problems in children, and to provide evidence-based support for early prevention strategies.

**Methods:**

PubMed, the Cochrane Library, Embase, Web of Science, SinoMed, CNKI, WanFang Data, and VIP were systematically searched from database inception to November 2025 for observational studies examining the association between breastfeeding and behavioral problems in children. Two reviewers independently screened the literature, extracted data, and assessed study quality. Meta-analysis was performed using Stata MP 18.0, and pooled odds ratios (ORs) with 95% confidence intervals (CIs) were calculated.

**Results:**

Ten studies involving 69,621 children were included, comprising four cohort studies and six cross-sectional studies. Breastfeeding was significantly associated with a lower risk of overall behavioral problems (OR = 0.66, 95% CI: 0.51–0.86, *p* = 0.002), internalizing behavioral problems (OR = 0.79, 95% CI: 0.69–0.89, *p* < 0.001), and externalizing behavioral problems (OR = 0.78, 95% CI: 0.69–0.89, *p* < 0.001). Substantial heterogeneity was observed for overall behavioral problems, whereas the pooled results for internalizing and externalizing behavioral problems were relatively stable. Subgroup analyses indicated that the association between breastfeeding and overall behavioral problems varied by study design and region. No significant association was observed in cohort studies or in studies conducted in China, whereas significant inverse associations were found in cross-sectional studies and in studies conducted in other countries. Longer breastfeeding duration (≥6 months) showed a stronger trend toward a lower risk, although the between-subgroup difference was not statistically significant.

**Conclusions:**

The findings suggest a potential association between breastfeeding and a lower risk of behavioral problems in children. However, the available evidence is limited, and residual confounding and publication bias cannot be excluded. Further high-quality prospective studies are needed to clarify the nature, magnitude, and potential causal mechanisms underlying these associations.

**Systematic Review Registration:**

https://www.crd.york.ac.uk/prospero/, identifier CRD420251233415.

## 1 Introduction

Mental health during childhood is crucial for healthy growth and development and has therefore become an important public health concern. The 2019 Global Burden of Disease study showed that the average prevalence of mental disorders among individuals aged 5–24 years worldwide was 11.63%. The corresponding prevalence estimates were 6.80%, 12.40%, and 13.96% among those aged 5–9, 10–14, and 15–19 years, respectively. These findings indicate that childhood and adolescence are critical periods during which mental disorders are highly prevalent (1). Because emotional regulation and behavioral control are still developing during these stages, children and adolescents are more vulnerable to a range of mental health problems (2). Among these, behavioral problems (BPs) are among the most common and prominent mental health concerns in childhood (3). In general, they refer to behavioral and emotional manifestations that exceed age-appropriate norms in frequency, intensity, or duration. Behavioral problems are commonly categorized into two broad dimensions: internalizing problems, which are characterized by inward emotional and cognitive difficulties such as anxiety, depression, and social withdrawal, and externalizing problems, which are characterized by outward behavioral dysregulation such as hyperactivity, aggression, and oppositional behavior (4). Previous studies have shown that, if not identified and addressed early, behavioral problems in childhood may be associated with impaired academic performance, difficulties in interpersonal relationships, and an increased risk of mental health and social functioning problems later in life (5, 6). These adverse effects may persist across developmental stages, affecting not only children's immediate well-being and developmental trajectories but also imposing a sustained burden on families and society (7). Against this background, identifying modifiable early-life factors that may be associated with behavioral problems has important implications for early prevention and child health promotion.

Breastfeeding is an important modifiable factor with significant public health implications for early childhood development (8). The World Health Organization recommends exclusive breastfeeding during the first 6 months of life, followed by continued breastfeeding alongside appropriate complementary feeding up to 2 years of age or beyond (9). The benefits of breastfeeding for children's physical health have been well established, including reduced risks of respiratory infections, obesity, and type 1 and type 2 diabetes (10). However, its long-term role in psychological and behavioral development has not been fully clarified, and the association between breastfeeding and behavioral problems remains controversial. Some studies have suggested that exclusive breastfeeding or longer breastfeeding duration may be associated with a lower risk of behavioral problems in children (11, 12), whereas others have reported no significant association (13, 14). These inconsistencies may be related to differences in study design, definitions of breastfeeding exposure, duration categorization, and the measurement of behavioral outcomes.

Although a previous systematic review explored this topic, it did not perform a meta-analysis and did not incorporate recently published studies (15). The available evidence only suggested that breastfeeding for 3–4 months or longer might be associated with fewer overall behavioral problems in children, whereas evidence regarding specific dimensions, such as internalizing and externalizing behavioral problems, remained insufficient (15). Observational studies remain the predominant source of evidence in breastfeeding research, given the ethical and practical limitations of randomized exposure allocation.

Therefore, the present study aimed to conduct a systematic review and meta-analysis of the most recent evidence to comprehensively evaluate the associations between breastfeeding and overall, internalizing, and externalizing behavioral problems in children, and to provide evidence for early prevention strategies and public health policies targeting child behavioral health.

## 2 Methods

This systematic review and meta-analysis of observational studies was conducted in accordance with the Preferred Reporting Items for Systematic Reviews and Meta-Analyses (PRISMA) 2020 statement (16). The study protocol was prospectively registered in the International Prospective Register of Systematic Reviews (PROSPERO; CRD420251233415).

### 2.1 Eligibility criteria

The eligibility criteria for this review have been structured according to the PICOS framework and are presented in Table 1.

**Table 1:** Eligibility criteria for study selection based on the PICOS framework.

Domain	Eligibility criteria
Population	*Inclusion criteria*: Studies including children aged < 18 years Studies conducted in general child populations, regardless of sex, birth weight, or geographical setting.
	Exclusion criteria: Studies focusing exclusively on children with major congenital anomalies, severe chronic diseases, or pre-existing neurodevelopmental/psychiatric conditions that may substantially influence breastfeeding practices or behavioral outcomes.
Intervention (Exposure)	*Inclusion criteria*: Breastfeeding, including any, exclusive, or partial breastfeeding Duration and timing of breastfeeding clearly described.
Comparator	*Inclusion criteria*: Never breastfed, formula-fed/artificially fed, or non-exclusively breastfed
Outcomes	*Inclusion criteria*: Studies reporting overall behavioral problems, internalizing behavioral problems, or externalizing behavioral problems assessed using validated measurement tools or recognized classifications of behavioral problems. Related subdomains were eligible if they could be classified under these broader behavioral dimensions.
	*Exclusion criteria*: Studies that did not report relevant behavioral outcomes or used outcome measures that could not be mapped to behavioral problems.
Study design	*Inclusion criteria*: Observational studies, including cohort studies and cross-sectional studies.
	*Exclusion criteria*: Randomized controlled trials, quasi-experimental studies, case-control studies, reviews, conference abstracts, case reports, editorials, letters, duplicate publications, and studies without accessible full texts. Studies that did not report extractable effect estimates or sufficient raw data to calculate ORs or RRs with 95% CIs were also excluded.

### 2.2 Search strategy

A systematic literature search was conducted independently by two reviewers in PubMed, the Cochrane Library, Embase, Web of Science, SinoMed, CNKI, WanFang Data, and VIP from database inception to November 2025. Additionally, the reference lists of included articles were manually screened to identify potentially eligible studies. Only studies published in English or Chinese were eligible for inclusion.

The search strategy combined controlled vocabulary terms and free-text terms. The main search terms included *Breast Feeding*, *Child*, *Teenager*, *Adolescent*, *Behavior Problem*, *Internalizing problems*, and *Externalizing problems*, as well as related Medical Subject Headings (MeSH) terms. The detailed search strategy for PubMed is provided in Supplementary Table S1.

### 2.3 Study selection

All retrieved records were imported into EndNote 21.5 for deduplication and screening. Two reviewers independently screened titles and abstracts, followed by full-text assessment of potentially eligible studies. Any disagreement was resolved through discussion, and a third reviewer was consulted when necessary. The study selection process was documented using a PRISMA flow diagram.

### 2.4 Data extraction

Data extraction was performed independently by two reviewers using a standardized data extraction form. Disagreements were resolved through discussion with a third reviewer.

The following information was extracted from each study: first author, publication year, country, study design, sample size, age of participants, breastfeeding-related data (including method, duration, and timing where available), type of behavioral outcome, assessment tools, adjusted covariates, methodological quality assessment results, and effect estimates used for meta-analysis.

### 2.5 Quality assessment

The methodological quality of included studies was assessed independently by two reviewers, and disagreements were resolved through discussion with a third reviewer.

Cohort studies were evaluated using the Newcastle–Ottawa Scale (NOS) (17), which assesses three domains: selection of study groups, comparability between groups, and outcome assessment. In the present study, NOS scores of 0–3, 4–6, and ≥7 were considered to indicate low, moderate, and high methodological quality, respectively.

Cross-sectional studies were assessed using the checklist recommended by the Agency for Healthcare Research and Quality (AHRQ) (18). Based on commonly used cut-off criteria in previous studies, AHRQ scores of 0–3, 4–7, and 8–11 were classified as low, moderate, and high methodological quality, respectively.

### 2.6 Certainty of evidence assessment

The certainty of evidence for each outcome was evaluated using the Grading of Recommendations Assessment, Development and Evaluation (GRADE) approach (19). Because all included evidence came from observational studies, the initial certainty rating was set as low. The certainty of evidence for each outcome was then judged by considering risk of bias, inconsistency, indirectness, imprecision, and publication bias. The methodological quality assessments based on the NOS and AHRQ tools were used primarily to inform the GRADE domain of risk of bias. The final certainty of evidence for each outcome was classified as high, moderate, low, or very low.

### 2.7 Statistical analysis

For dichotomous outcomes, pooled effect estimates were expressed as odds ratios (ORs) with 95% confidence intervals (CIs). When both crude and adjusted effect estimates were reported, the most fully adjusted estimate was extracted for meta-analysis. For studies reporting risk ratios (RRs) with 95% CIs, RR was treated as approximately equivalent to OR, considering that only one included study used RR as the effect measure and that the event rate was relatively low in most included studies, consistent with commonly used approaches in previous meta-analyses of low-incidence outcomes (20). When a single study reported multiple effect estimates across different breastfeeding duration categories, only one estimate was included in each pooled analysis to avoid double counting. For the main analysis, the estimate corresponding to the longest breastfeeding duration was extracted, representing the maximum cumulative exposure. For subgroup analyses by breastfeeding duration, the estimate closest to the predefined categories (<6 months or ≥6 months) was selected.

Overall behavioral problems, internalizing behavioral problems, and externalizing behavioral problems were analyzed as separate outcomes. Given the anticipated clinical and methodological heterogeneity across studies in terms of study design, breastfeeding exposure definitions, duration categorization, behavioral assessment tools, and confounding adjustment, a random-effects model was prespecified as the primary analytical model. Statistical heterogeneity was assessed using Cochran's *Q-*test and the *I*^2^ statistic. A *Q-*test with *p* < 0.10 or an *I*^2^ value >50% was considered to indicate substantial heterogeneity. When heterogeneity was low (*Q*-test *p* ≥ 0.10 and *I*^2^ ≤ 50%), a fixed-effect model was additionally applied as a supplementary analysis to evaluate the robustness of the pooled estimates.

For outcomes with substantial heterogeneity, subgroup analyses were performed according to study design (cohort vs. cross-sectional), study region (China vs. other countries), and breastfeeding duration (<6 months vs. ≥6 months) to explore potential sources of heterogeneity. Sensitivity analyses were conducted using a leave-one-out approach to assess the robustness of the pooled results.

When a meta-analysis included at least 10 studies, publication bias was assessed visually using funnel plots and quantitatively using Egger's regression test. Except for heterogeneity testing, a two-sided *p* value <0.05 was considered statistically significant. All statistical analyses were performed using Stata MP 18.0.

## 3 Results

### 3.1 Study selection

**Figure 1:**
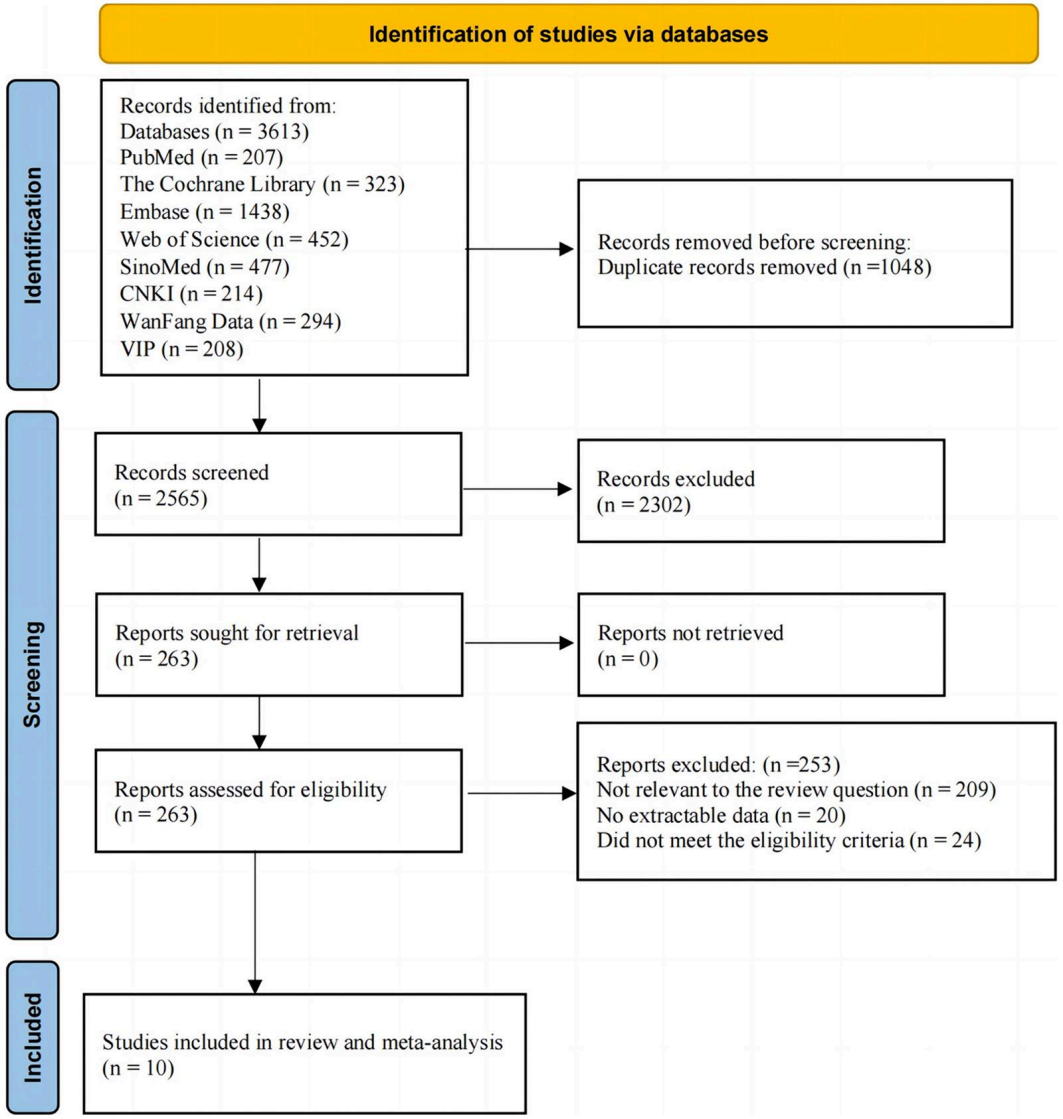
The PRISMA flow diagram.

A total of 3,613 records were identified from the eight databases. After removal of 1,048 duplicates, 2,565 records remained for title and abstract screening. Of these, 2,302 records were excluded, and 263 articles were assessed in full text. After full-text review, 253 articles were excluded, including 209 that were not relevant to the review topic, 20 with insufficient data for extraction, and 24 that did not meet the eligibility criteria. Ultimately, 10 studies were included in the systematic review and meta-analysis. The study selection process is presented in Figure 1.

### 3.2 Study characteristics and quality assessment

Ten observational studies published between 2011 and 2025 were included, involving a total of 69,621 children from seven countries, including China, the United States, the United Kingdom, Ethiopia, Korea, Ireland, and Egypt. Of these, nine studies (21–29) were published in English and one (30) in Chinese. Four studies (21–23, 30) used a cohort design, and six (24–29) used a cross-sectional design. The age of participants ranged from 0.5 to 11 years.

With regard to exposure definitions, most studies compared breastfeeding with never breastfeeding, and some further distinguished exclusive breastfeeding. The categorization of breastfeeding duration varied considerably across studies, including <2 months, 2–4 months, >4 months, <3 months, ≥3 months, <6 months, >6 months, and 7–12 months, 13–24 months, or ≥25 months. Some studies (25, 29) did not further stratify breastfeeding duration.

Behavioral outcomes were categorized as overall behavioral problems, internalizing behavioral problems, and externalizing behavioral problems. Some studies also reported specific subdomains, including emotional symptoms, peer problems, conduct problems, and hyperactivity/inattention. The main assessment tools included the Revised Parent's Rutter Scale (RPRS) (22), the Strengths and Difficulties Questionnaire (SDQ) (21, 23, 26, 28), the Child Behavior Checklist (CBCL) (27, 29, 30), Parent-reported questionnaire from the National Survey of Children's Health (NSCH) (24), and the Bayley-III scale (25). Detailed study characteristics are presented in Table 2.

**Table 2:** Characteristics of the included studies.

Author	Year	Country	Design	Sample size	Age at behavioral assessment (years)	Breastfeeding EG/RG	Duration category	Outcome domain	Assessment tool	Adjusted covariates
Kwok et al. (22)	2013	China	Cohort	5,598	11	EBF vs. NBF	<3 months;	Overall behavioral problems;	RPRS	1,2,3,4,13,14,15,16,17
							≥3 months	Conduct problems;		
								Emotional symptoms;		
								Hyperactivity problems		
Lind et al. (23)	2014	USA	Cohort	1,442	6	BF + EBF vs. NBF	BF < 6 months;	Overall behavioral problems;	SDQ	1,3,4,14,16,18,19,20,21,32,33,34,35,36
							BF ≥ 6 months with EBF < 3 months;	Emotional symptoms;		
								Peer problems;		
							BF ≥ 6 months with EBF ≥ 3 months	Conduct problems;		
								Hyperactivity/inattention		
Heikkilä et al. (21)	2011	UK	Cohort	9,525	5	BF vs. NBF	<2 months;	Overall behavioral problems;	SDQ	4,5,6,14,16,18,19,22,27,37,38
							2–4 months;	Emotional symptoms;		
							≥4 months	Peer problems;		
								Conduct problems;		
								Hyperactivity/inattention		
Qiang et al. (30)	2022	China	Cohort	305	4	BF vs. NBF	<3 months;	Overall behavioral problems;	CBCL	1,3,7,14,16,18,37,39,40,41
							4–5 months;	Internalizing behavioral problems;		
							6–11 months;	Externalizing behavioral problems		
							≥12 months			
Metwally et al. (25)	2016	Egypt	Cross-sectional	655	0.5–1	BF vs. NBF	Not reported	Socioemotional development	Bayley-III	4,8,9,14,15
Kiros et al. (26)	2025	Ethiopia	Cross-sectional	606	2–6	BF vs. NBF	≥6 months	Overall behavioral problems	SDQ	2,7,10,14,15,19,23,24,25,26
Park et al. (29)	2014	Korea	Cross-sectional	874	8–11	BF vs. NBF	Not reported	Overall behavioral problems;	CBCL	1,2,11,16,17,28
								Internalizing behavioral problems;		
								Externalizing behavioral problems		
Pan et al. (24)	2025	China	Cross-sectional	40,280	3–5	BF vs. NBF	<6 months;	Psychological problems;	NSCH	1,2,3,4,7,13,14,16,20,22,29
							7–12 months;	Behavioral problems		
							13–24 months;			
							≥25 months			
Huang et al. (27)	2019	China	Cross-sectional	1,979	6–11	BF vs. NBF	<6 months;	Internalizing behavioral problems;	CBCL	1,2,14,16,29,30,37,40,41,42,43
							≥6 months			
								Externalizing behavioral problems		
Reynolds et al. (28)	2014	Ireland	Cross-sectional	8,357	9	BF vs. NBF	≤10 weeks;	Overall behavioral problems	SDQ	14,16,17,18,19,22
							11–25 weeks;			
							≥26 weeks			

EG, exposure group, RG, reference group, BF, breastfeeding; EBF, exclusive breastfeeding; NBF, never breastfed, formula-fed, or artificially fed.

**Adjusted covariates**: 1, sex;2, age;3, birth weight;4, birth order/number of siblings;5, history of neonatal intensive care unit admission;6, age at entry into childcare and type of childcare;7, preterm birth/gestational age;8, timing of complementary feeding introduction;9, serum magnesium level;10, physical health status;11, intelligence quotient (IQ);12, obesity;13, paternal/maternal reproductive age;14, paternal/maternal educational level;15, paternal/maternal employment status;16, household income;17, maternal place of birth;18, paternal/maternal age;19, marital status;20, ethnicity/race;21, maternal smoking during the first postpartum year;22, maternal physical and mental health;23, type of primary caregiver;24, caregiver history of substance abuse;25, caregiver educational level;26, caregiver mental health;27, residential area;28, maternal IQ;29, household smoking exposure;30, parenting style;31, family structure;32, gestational week;33, maternal and infant nutritional supplementation during the postpartum period;34, participation in activities;35, pre-pregnancy body mass index (BMI);36, postpartum depression;37, smoking/alcohol use during pregnancy;38, mother–infant attachment;39, first-trimester BMI;40, mode of delivery;41, medication use during pregnancy;42, x-ray exposure during pregnancy;43, adverse pregnancy history.

**Assessment tools:** RPRS, Revised Parent's Rutter Scale; SDQ, Strengths and Difficulties Questionnaire; CBCL, Child Behavior Checklist; Bayley-III, Bayley Scales of Infant and Toddler Development, Third Edition; NSCH, Parent-reported questionnaire from the National Survey of Children's Health.

The methodological quality of the included studies was assessed using the Newcastle–Ottawa Scale (NOS) for cohort studies and the Agency for Healthcare Research and Quality (AHRQ) checklist for cross-sectional studies. Quality scores ranged from 4 to 8. Four studies (21, 24, 26, 30) were rated as high quality, whereas six (22, 23, 25, 27–29) were rated as moderate quality. The detailed quality assessment results are provided in Supplementary Tables S2 and S3.

### 3.3 Certainty of evidence

The certainty of evidence for each outcome was evaluated using the GRADE approach. Because all included studies were observational in design, the initial certainty of evidence for all outcomes was rated as low.

For overall behavioral problems, the certainty of evidence was further downgraded by two levels to very low. One downgrade was applied for risk of bias because breastfeeding exposure was commonly based on parental recall, behavioral outcomes were largely assessed using subjective parent- or teacher-reported measures, and adjustment for important confounders, such as maternal mental health, parenting characteristics, and socioeconomic factors, was inconsistent across studies. A second downgrade was applied for inconsistency because substantial heterogeneity was observed among studies (*I*^2^ = 62.88%, *p* = 0.009), and subgroup analyses suggested variation according to study design and geographic region. No serious indirectness was identified because the included populations, exposures, and outcomes were broadly consistent with the review question. In addition, the pooled confidence interval did not cross the null value, indicating no serious imprecision. Publication bias was not formally assessed because the number of included studies was limited.

In contrast, the certainty of evidence for both internalizing behavioral problems and externalizing behavioral problems remained low. The pooled estimates for these outcomes were relatively consistent, and statistical heterogeneity was low (*I*^2^ = 21.60% and 1.76%, respectively). However, because all included evidence was observational, residual confounding and potential measurement bias could not be completely excluded. Furthermore, methodological variability remained across studies, including differences in breastfeeding exposure definitions and behavioral outcome assessments.

Overall, the GRADE assessment indicated that the current evidence supporting an association between breastfeeding and behavioral problems in children remains limited, particularly for overall behavioral problems. Therefore, although breastfeeding was associated with lower risks of behavioral problems in the pooled analyses, the findings should be interpreted cautiously. Detailed certainty ratings for each outcome are presented in Table 3.

**Table 3:** Quality of evidence assessment using the GRADE approach.

Outcome	No. of studies	Study design	Pooled effect estimate	Risk of bias	Inconsistency	Indirectness	Imprecision	Publication bias	Certainty of evidence	Explanation
Overall behavioral problems	8	Observational studies	OR = 0.66, 95% CI: 0.51–0.86	Serious	Serious	Not serious	Not serious	Not assessed;	⊕◯◯◯ Very low	Most included studies were observational, with limitations in exposure ascertainment, subjective outcome assessment, and confounding control. Substantial heterogeneity was present (I² = 62.88%), and variation across study design, study region, breastfeeding exposure definitions, and behavioral assessment methods further reduced confidence in the pooled estimate.
								Not downgraded		
Internalizing behavioral problems	7	Observational studies	OR = 0.79, 95% CI: 0.69–0.89	Not serious	Not serious	Not serious	Not serious	Not assessed;	⊕⊕◯◯ Low	Findings were relatively consistent, with low heterogeneity (I² = 21.60%). However, all included studies were observational, and residual confounding, subjective outcome assessment, and methodological variability in exposure and outcome definitions could not be fully excluded.
								Not downgraded		
Externalizing behavioral problems	7	Observational studies	OR = 0.78, 95% CI: 0.69–0.89	Not serious	Not serious	Not serious	Not serious	Not assessed;	⊕⊕◯◯ Low	Results were stable, with minimal heterogeneity (I² = 1.76%). However, all included studies were observational, and residual confounding, subjective outcome assessment, and non-uniform exposure and outcome definitions reduced confidence in the pooled estimate.
								Not downgraded		

The certainty of evidence was assessed using the Grading of Recommendations Assessment, Development and Evaluation (GRADE) approach. Because all included studies were observational in design, the initial certainty of evidence was rated as low. Evidence was downgraded according to risk of bias, inconsistency, indirectness, imprecision, and publication bias. Publication bias was not formally assessed for all outcomes because of the limited number of studies and the lack of complete independence among some comparisons; therefore, no downgrade was applied for this domain.

### 3.4 Meta-analysis results

#### 3.4.1 Meta-analysis of overall behavioral problems

**Figure 2:**
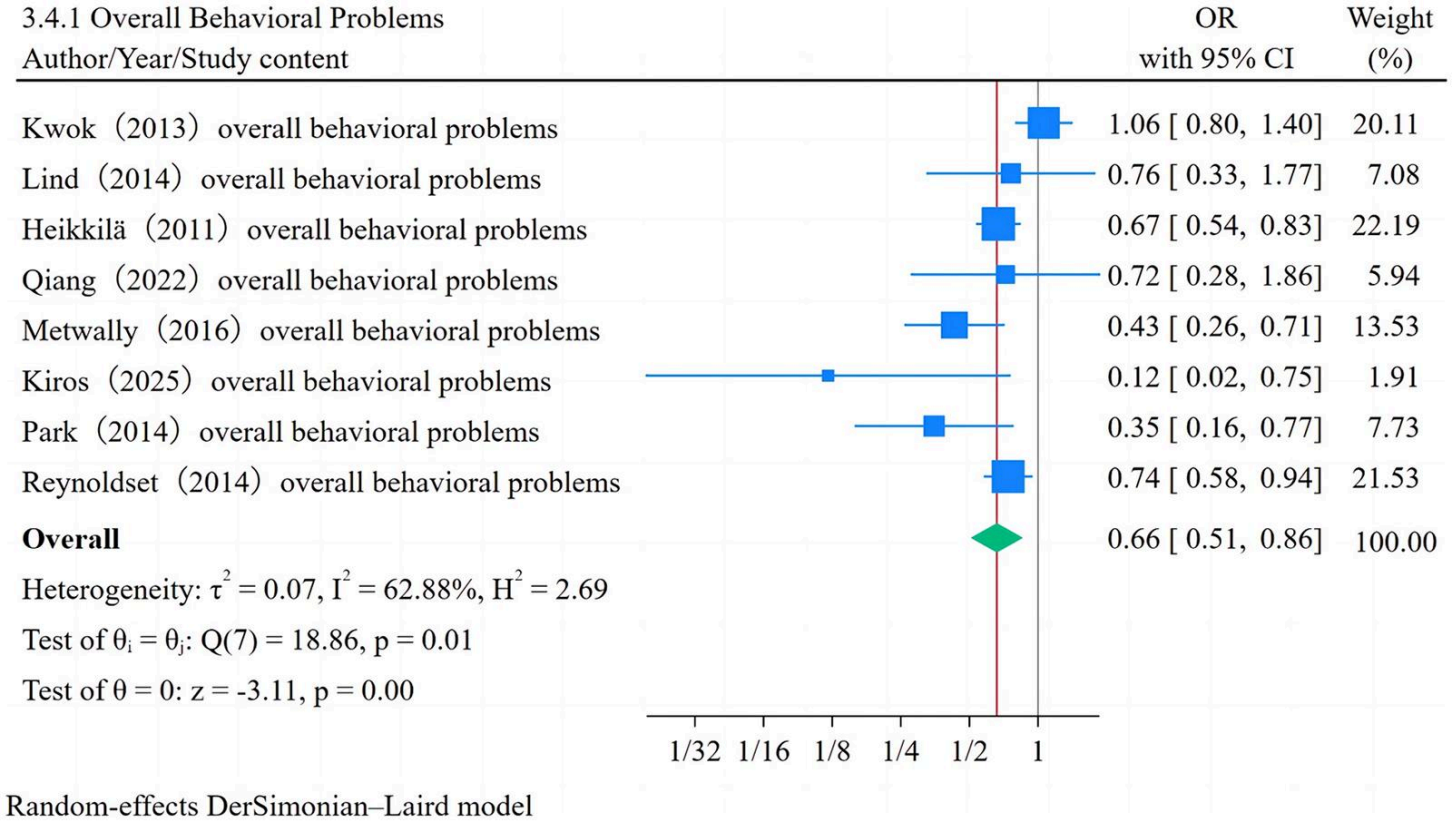
Forest plot of the association between breastfeeding and overall behavioral problems in children.

Eight studies (8 independent comparisons) were included in the meta-analysis of overall behavioral problems. Substantial heterogeneity was observed across studies (Q = 18.86, *p* = 0.009; *I*^2^ = 62.88%); therefore, a random-effects model was used. The pooled analysis showed that breastfeeding was significantly associated with a lower risk of overall behavioral problems in children compared with never breastfeeding (OR = 0.66, 95% CI: 0.51–0.86, *p* = 0.002). The forest plot is shown in Figure 2.

#### 3.4.2 Meta-analysis of internalizing behavioral problems

**Figure 3:**
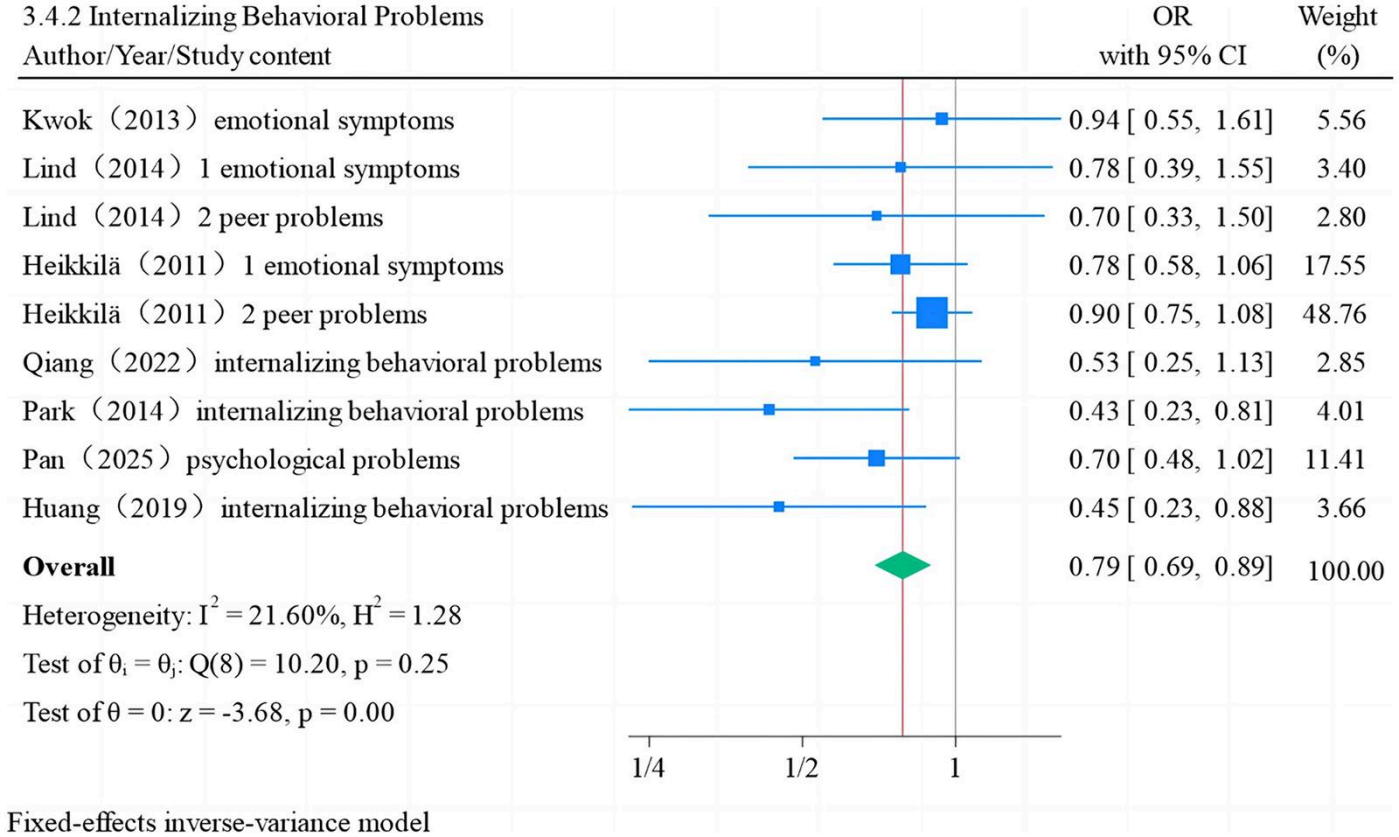
Forest plot of the association between breastfeeding and internalizing behavioral problems in children.

Seven studies (9 independent comparisons) were included in the meta-analysis of internalizing behavioral problems. Heterogeneity was low (Q = 10.20, *p* = 0.251; *I*^2^ = 21.60%); therefore, a fixed-effect model was used. The pooled OR was 0.79 (95% CI: 0.69–0.89, *p* < 0.001), indicating that breastfeeding was significantly associated with a lower risk of internalizing behavioral problems. The forest plot is shown in Figure 3.

#### 3.4.3 Meta-analysis of externalizing behavioral problems

**Figure 4:**
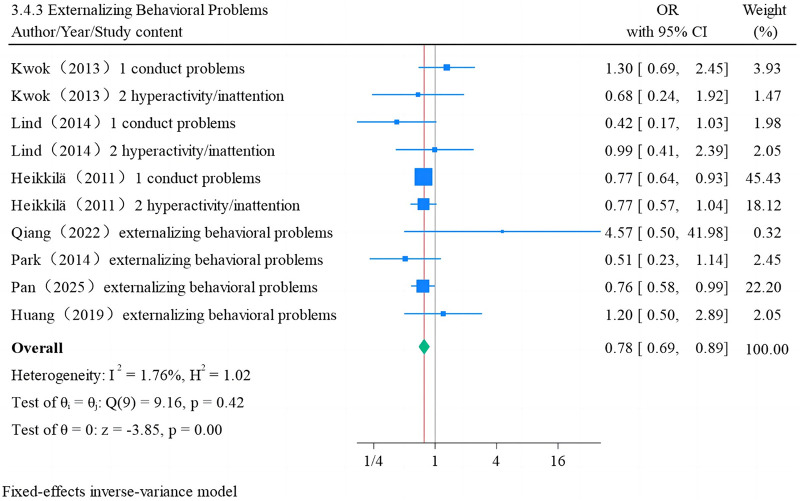
Forest plot of the association between breastfeeding and externalizing behavioral problems in children.

Seven studies (10 independent comparisons) were included in the meta-analysis of externalizing behavioral problems. Heterogeneity was low (Q = 9.16, *p* = 0.423; *I*^2^ = 1.76%); therefore, a fixed-effect model was used. The pooled OR was 0.78 (95% CI: 0.69–0.89, *p* < 0.001), indicating that breastfeeding was significantly associated with a lower risk of externalizing behavioral problems. The forest plot is shown in Figure 4.

### 3.5 Subgroup analysis

Because substantial heterogeneity was identified in the meta-analysis of overall behavioral problems, subgroup analyses were performed according to study design, study region, and breastfeeding duration. Detailed subgroup analysis results are shown in Table 4.

**Table 4:** Subgroup analyses of the association between breastfeeding and overall behavioral problems in children.

Subgroup variable	Category	No. of studies	Pooled OR (95% CI)	*p* value	Model	I² (%)	*P* for heterogeneity	*p* for subgroup difference
Study design	Cohort	4	0.81 (0.60–1.11)	0.188	Random-effects	54.1	0.088	0.077
	Cross-sectional	4	0.47 (0.27–0.79)	0.005	Random-effects	67.9	0.025	
Region	China	2	1.03 (0.79–1.34)	0.843	Fixed-effect	0.0	0.443	<0.001
	Other countries	6	0.65 (0.56–0.75)	<0.001	Fixed-effect	48.2	0.086	
Breastfeeding duration	<6 months	4	0.95 (0.78–1.15)	0.558	Random-effects	63.6	0.007	0.415
	≥6 months	3	0.82 (0.61–1.09)	0.167	Fixed-effect	9.1	0.358	

When stratified by study design, the between-subgroup difference was not statistically significant (*p* = 0.077). The pooled OR was 0.81 (95% CI: 0.60–1.11) for cohort studies and 0.47 (95% CI: 0.27–0.79) for cross-sectional studies.

When stratified by study region, the between-subgroup difference was statistically significant (*p* < 0.001). The pooled OR was 1.03 (95% CI: 0.79–1.34) for studies conducted in China and 0.65 (95% CI: 0.56–0.75) for studies conducted in other countries.

When stratified by breastfeeding duration, the between-subgroup difference was not statistically significant (*p* = 0.415). The pooled OR was 0.95 (95% CI: 0.78–1.15) for breastfeeding duration <6 months and 0.82 (95% CI: 0.61–1.09) for breastfeeding duration ≥6 months.

### 3.6 Sensitivity analysis

Sensitivity analyses were conducted using a leave-one-out approach for the meta-analyses of overall behavioral problems, internalizing behavioral problems, and externalizing behavioral problems. After sequential omission of each study, the direction of the pooled effect estimates remained unchanged, the magnitude of the pooled estimates did not materially change, and all 95% CIs remained on the same side of the null value. These findings suggested that the pooled results were robust and were not driven by any single study. The results of the sensitivity analyses are shown in Supplementary Figures S1–S3.

### 3.7 Publication bias

Formal funnel plot assessment was not performed for overall behavioral problems or internalizing behavioral problems because only 8 and 9 independent comparisons were included, respectively, which was considered insufficient for a stable assessment of funnel plot asymmetry. For externalizing behavioral problems, although 10 independent comparisons were available, some effect estimates were derived from the same study and therefore did not fully meet the assumption of independence. Accordingly, only an exploratory funnel plot was generated. Visual inspection suggested some asymmetry, with one comparison located outside the main distribution of studies. This pattern may reflect small-study effects or between-study variation; however, no firm conclusion regarding publication bias could be drawn. The funnel plot is presented in Supplementary Figure S4.

Given the limited number of studies across the three outcomes and the lack of complete independence among some comparisons, Egger's regression test was not performed.

## 4 Discussion

The association between breastfeeding and child behavioral problems remains inconclusive. In this systematic review and meta-analysis of 10 observational studies involving 69,621 children, breastfeeding was associated with lower risks of overall, internalizing, and externalizing behavioral problems. However, these findings should be interpreted cautiously because behavioral problems are broad and multidimensional constructs, and the included studies differed in behavioral outcome definitions, assessment tools, informants, and outcome thresholds. Thus, the pooled analyses should be understood as a synthesis of related but not fully equivalent behavioral constructs rather than as evidence for a single uniformly defined outcome. Consistent with this, substantial heterogeneity was observed for overall behavioral problems, whereas the pooled results for internalizing and externalizing behavioral problems were relatively more stable. According to the GRADE assessment, the certainty of evidence was very low for overall behavioral problems and low for internalizing and externalizing behavioral problems, mainly because of heterogeneity, residual confounding, and potential measurement bias. Therefore, although the present findings suggest a potential protective association between breastfeeding and child behavioral outcomes, the available evidence remains limited.

For overall behavioral problems, breastfeeding was associated with a lower pooled risk compared with never breastfeeding. This finding is generally consistent with that of the previous systematic review, further suggesting that breastfeeding may be associated with child behavioral development (15). In addition, the present study incorporated more recently published evidence, thereby providing a more up-to-date synthesis of the available literature. However, heterogeneity was substantial, and the pooled findings should therefore be interpreted with caution. Subgroup analyses suggested that regional differences were the most prominent source of between-study variation, while differences by study design and breastfeeding duration showed directional variation but did not reach statistical significance. This pattern is likely to reflect differences across countries and settings in breastfeeding support policies, parenting practices, sociocultural context, and approaches to identifying behavioral problems.

Importantly, the apparently stronger association observed in cross-sectional studies should not be interpreted as stronger evidence. Cross-sectional studies are more vulnerable to recall bias, same-source measurement bias, reverse causation, and residual confounding because breastfeeding exposure and behavioral outcomes are often ascertained retrospectively and measured concurrently. By contrast, cohort studies are generally more informative for temporal ordering and causal interpretation, even though the pooled association in the present subgroup analysis was not statistically significant. In line with this interpretation, Belfort et al. (31) reported that the association between longer breastfeeding duration and behavioral outcomes in middle childhood was attenuated after more extensive adjustment for maternal characteristics, family environment, maternal mental health, and socioeconomic factors. Therefore, the apparently stronger associations in cross-sectional studies and in studies conducted outside China are more likely to reflect differences in susceptibility to bias, study context, and outcome operationalization than direct evidence of a stronger underlying association in specific populations. This interpretation is also consistent with the GRADE assessment, in which the certainty of evidence for overall behavioral problems was rated as very low.

It is also noteworthy that the pooled effect estimate for breastfeeding duration ≥6 months was lower than that for breastfeeding duration <6 months, suggesting a potentially stronger association with longer breastfeeding duration, although the between-subgroup difference was not statistically significant. This pattern is generally consistent with some previous studies, which suggest that longer breastfeeding duration may be associated with fewer behavioral problems (11, 32). However, not all studies have supported a progressively stronger association with increasing duration. Some more recent findings suggest that the potential benefit of breastfeeding may be concentrated in the first months of life, with no clear additional benefit beyond a certain duration (33). This pattern is likely to reflect that the association between breastfeeding and child behavioral development may depend not only on duration itself but also on the intensity of feeding and related mother–infant interaction during early life. At present, the available evidence does not support a clear optimal duration threshold. This inconsistency may be associated with methodological heterogeneity across studies, particularly in the definition of breastfeeding exposure and the assessment of behavioral outcomes. Breastfeeding exposure was not defined uniformly, including contrasts based on any breastfeeding, exclusive breastfeeding, and different duration thresholds. Behavioral outcomes were also assessed using different instruments and subdomains, including the Rutter Scale, SDQ, CBCL, Bayley-III, and NSCH-based parent-reported classifications, with varying informants and cut-off definitions. Together, these differences may have reduced the comparability of effect estimates and contributed to the substantial between-study heterogeneity.

In the dimension-specific analyses, breastfeeding was further associated with a lower risk of internalizing behavioral problems in children. This finding is generally consistent with the overall direction of previous observational evidence. For example, Meng et al. (34) reported in a cohort study that exclusive breastfeeding during the first 6 months and longer breastfeeding duration were associated with lower levels of internalizing behavioral problems. Several mechanisms may underlie this association. From a biological perspective, breast milk contains long-chain polyunsaturated fatty acids (LCPUFAs), including arachidonic acid (AA), docosahexaenoic acid (DHA), and eicosapentaenoic acid (EPA), which are important structural components of the developing brain and nervous system. These nutrients are involved in synapse formation and neurotransmission relevant to emotional regulation (35) and may therefore be associated with a lower risk of later anxiety- and depression-related symptoms.

However, the association between breastfeeding and children's mental health may not be explained by nutritional mechanisms alone. Psychosocial pathways may also play an important role. Liu et al. (36) found that children who were exclusively breastfed and experienced positive mother–infant interaction had the lowest levels of later internalizing behavioral problems. Eye contact, skin-to-skin contact, and sucking during feeding may be associated with increased oxytocin release and reduced cortisol levels in both mother and infant (37, 38). Such neuroendocrine regulation may support secure attachment, attenuate stress responses, and promote socioemotional development (39). Previous studies have also suggested that reduced postpartum depressive symptoms and improved parent–child relationship quality may partly mediate the association between breastfeeding and internalizing outcomes (40). Taken together, these findings suggest that any potential association between breastfeeding and internalizing behavioral problems is likely to reflect the combined influence of nutritional, neuroendocrine, and psychosocial factors.

Similar to the findings for internalizing behavioral problems, breastfeeding was also associated with a lower risk of externalizing behavioral problems, with an approximately 22% reduction in the pooled risk estimate. This finding is generally consistent with part of the existing literature. For example, Hayatbakhsh et al. (41) reported that longer breastfeeding duration was associated with fewer social problems, attention problems, and aggressive behaviors during adolescence. It should be noted, however, that many previous studies have focused on more specific neurobehavioral phenotypes within the externalizing spectrum, particularly attention-deficit/hyperactivity disorder (ADHD). Julvez (42), Jallow (43), and Zeng (44) reported that breastfeeding was associated with fewer hyperactivity/inattention symptoms or a lower risk of ADHD. Compared with these studies, which focused on more specific phenotypes such as hyperactivity, impulsivity, and attention deficits, the present study evaluated a broader category of externalizing behavioral problems. Therefore, the current literature more directly supports a potential association between breastfeeding and the attention/hyperactivity dimension of externalizing behavior, whereas the present findings suggest that this association may extend to broader externalizing outcomes. Whether this association is comparable across all externalizing subdomains remains to be clarified.

Several mechanisms may underlie this association. First, breast milk is rich in AA, DHA, and EPA, which are important for fetal and infant brain development (45). Early deficiency of these nutrients may be associated with an increased risk of later externalizing behavioral problems, and some of these behavioral characteristics may persist into adolescence (39). Neuroimaging studies have further suggested that this association may involve neural pathways related to behavioral regulation, including frontostriatal circuits (35). Second, bioactive microbiota in breast milk may contribute to the establishment of infant gut microbial homeostasis and may influence brain development and behavior through the microbiota–gut–brain axis (46). These pathways may be associated with hyperactivity, impulsivity, and attentional dysregulation. However, this association may not be explained by biological pathways alone. Some studies (47) support a mediating role for mother–infant relationship quality and attachment in the association between breastfeeding and later behavioral outcomes. Taken together, these findings suggest that the association between breastfeeding and externalizing behavioral problems is likely to reflect both biological and psychosocial processes rather than a single explanatory pathway.

This review has several strengths. First, we systematically searched multiple English- and Chinese-language databases, thereby expanding the coverage of the available evidence and incorporating recently published studies. Second, given that direct random allocation of breastfeeding exposure is limited by ethical and practical considerations, observational evidence remains the main source for evaluating this question, and the focus of the present review was therefore appropriate. Third, this study examined not only overall behavioral problems but also internalizing behavioral problems and externalizing behavioral problems separately, which allowed a more refined evaluation of potential differences across behavioral dimensions. Finally, adjusted effect estimates were extracted whenever possible, which may have reduced the influence of measured confounding factors and improved the robustness of the pooled estimates.

### 4.1 Study limitations and future directions

Several limitations should be acknowledged. Behavioral outcomes were assessed largely through parent- or teacher-reported measures, which may have introduced information bias. Definitions of breastfeeding exposure, duration categories, and behavioral outcomes were not fully consistent across studies, and breastfeeding duration was commonly collected through parental recall, which may have introduced exposure misclassification. Confounding adjustment also varied across studies, and important factors such as maternal mental health, family environment, parenting characteristics, and socioeconomic conditions were not controlled consistently. In addition, follow-up completeness may have differed across longitudinal studies. Because the evidence base was observational, causal inference remains limited, and the stronger pooled associations observed in cross-sectional studies than in cohort studies may reflect greater susceptibility to recall bias, reverse causation, and unmeasured confounding rather than stronger evidence of an underlying effect. These limitations reduced confidence in the pooled estimates and contributed to the low or very low GRADE ratings across outcomes. Therefore, the findings should be interpreted cautiously.

Future high-quality prospective studies with standardized exposure definitions, improved follow-up, multi-informant assessment, and more rigorous confounding control are needed to clarify these associations.

## 5 Conclusions

In conclusion, the findings suggest a potential association between breastfeeding and a lower risk of behavioral problems in children. However, residual confounding and publication bias cannot be excluded. Given that the available evidence is derived entirely from observational studies and that the certainty of evidence ranged from very low to low across outcomes, causal inference remains limited. From a practical perspective, mothers who are able and willing to breastfeed should continue to receive support for breastfeeding initiation and continuation in accordance with current public health recommendations. However, the present findings should not be interpreted as evidence for a specific breastfeeding duration threshold to prevent behavioral problems. Further high-quality prospective studies are needed to clarify the nature, magnitude, and potential causal mechanisms underlying these associations.

## Data Availability

The original contributions presented in the study are included in the article/Supplementary Material, further inquiries can be directed to the corresponding author.
