# Supplementary material for: Association between breastfeeding and lower risk of behavioral problems in children: a systematic review and meta-analysis
Source: Front Pediatr. 2026 Jun 12;14:1867649. doi: 10.3389/fped.2026.1867649 (PMC13303373; doi:10.3389/fped.2026.1867649)
Supplement: Supplementary file 1 [file Supplementaryfile1.docx]

**Supplementary package**

**Supplementary Table S1. PubMed** **search strategy.**

| Step | Search strategy |
| --- | --- |
| #1 | ("Breast Feeding"[MeSH]) OR (Breast Feeding[Title/Abstract] OR Breast Feedings[Title/Abstract] OR Breast Fed[Title/Abstract] OR Breastfed[Title/Abstract] OR Chestfeeding[Title/Abstract] OR Breastfeeding[Title/Abstract] OR Breast Feeding, Exclusive[Title/Abstract] OR Exclusive Breast Feeding[Title/Abstract] OR Breastfeeding, Exclusive[Title/Abstract] OR Exclusive Breastfeeding[Title/Abstract] OR Wet Nursing[Title/Abstract] OR Milk Sharing[Title/Abstract] OR Sharing, Milk[Title/Abstract]) |
| #2 | (("Child"[Mesh]) OR (((((((child[Title/Abstract]) OR (teenager[Title/Abstract])) OR (youth[Title/Abstract])) OR (juvenile[Title/Abstract])) OR (children[Title/Abstract])) OR (minor[Title/Abstract])) OR (minors[Title/Abstract]))) OR (("Adolescent"[Mesh]) OR ((((((((((adolescent[Title/Abstract]) OR (Adolescents[Title/Abstract])) OR (Adolescence[Title/Abstract])) OR (Teens[Title/Abstract])) OR (Teen[Title/Abstract])) OR (Teenagers[Title/Abstract])) OR (Teenager[Title/Abstract])) OR (Youth[Title/Abstract])) OR (Youths[Title/Abstract])) OR (Adolescents, Female[Title/Abstract] OR Adolescent, Female[Title/Abstract] OR Female Adolescent[Title/Abstract] OR Female Adolescents[Title/Abstract] OR Adolescents, Male[Title/Abstract] OR Adolescent, Male[Title/Abstract] OR Male Adolescent[Title/Abstract] OR Male Adolescents [Title/Abstract]))) |
| #3 | (((("Problem Behavior"[Mesh]) OR (Behavior, Problem[Title/Abstract] OR Behaviors, Problem[Title/Abstract] OR Problem Behaviors[Title/Abstract] OR Behavioral Problem[Title/Abstract] OR Behavioral Problems[Title/Abstract] OR Problem, Behavioral[Title/Abstract] OR Problems, Behavioral[Title/Abstract] OR Disruptive Behavior[Title/Abstract] OR Behavior, Disruptive[Title/Abstract] OR Behaviors, Disruptive[Title/Abstract] OR Disruptive Behaviors[Title/Abstract] OR Dysfunctional Behavior, Psychology[Title/Abstract] OR Behavior, Psychology Dysfunctional[Title/Abstract] OR Behaviors, Psychology Dysfunctional[Title/Abstract] OR Dysfunctional Behaviors, Psychology[Title/Abstract] OR Psychology Dysfunctional Behavior[Title/Abstract] OR Psychology Dysfunctional Behaviors[Title/Abstract])) OR (("Conduct Disorder"[Mesh]) OR (Conduct Disorders[Title/Abstract] OR Callous-Unemotional Traits[Title/Abstract] OR Callous-Unemotional Trait[Title/Abstract] OR Callous Unemotional Traits [Title/Abstract]))) OR (("Mental Health"[Mesh]) OR (Health, Mental[Title/Abstract] OR Mental Hygiene[Title/Abstract] OR Hygiene, Mental[Title/Abstract]))) OR ("Emotional and behavioral problem"[Title/Abstract] OR "Emotional and behavioral problems"[Title/Abstract] OR Emotional Problems[Title/Abstract] OR Emotional Difficulties[Title/Abstract] OR Emotional Disorders[Title/Abstract] OR Behavioral Difficulties[Title/Abstract] OR Behavioral Disorders[Title/Abstract] OR "Internalizing and externalizing problem"[Title/Abstract] OR "Internalizing and externalizing problems"[Title/Abstract] OR Internalizing problems[Title/Abstract] OR Externalizing problems[Title/Abstract]) |
| #4 | #1 AND #2 AND #3 |

**Supplementary Table S2. Quality assessment of included cohort studies.**

| Study | Representativeness of exposed cohort | Selection of non-exposed cohort | Ascertainment of exposure | Outcome assessment at study start | Comparability of cohorts | Outcome assessment | Adequacy of follow-up | Completeness of follow-up | Total score | Quality rating |
| --- | --- | --- | --- | --- | --- | --- | --- | --- | --- | --- |
| Kwok et al., 2013 | 1 | 1 | 0 | 1 | 2 | 0 | 1 | 0 | 6 | Moderate |
| Lind et al., 2014 | 0 | 1 | 0 | 1 | 2 | 0 | 1 | 0 | 5 | Moderate |
| Heikkilä et al., 2011 | 1 | 1 | 1 | 1 | 2 | 0 | 1 | 0 | 8 | High |
| Qiang et al., 2022 | 1 | 1 | 1 | 1 | 2 | 0 | 1 | 0 | 7 | High |

**Notes:** Quality assessment was performed using the Newcastle–Ottawa Scale (NOS) for cohort studies. Scores range from 0–3 = low quality, 4–6 = moderate quality, and ≥7 = high quality. Each column represents the key domain evaluated in the NOS, including selection, comparability, and outcome assessment.

**Supplementary Table S3. Quality assessment of included cross-sectional studies.**

| Study | ① | ② | ③ | ④ | ⑤ | ⑥ | ⑦ | ⑧ | ⑨ | ⑩ | ⑪ | Total score | Rating |
| --- | --- | --- | --- | --- | --- | --- | --- | --- | --- | --- | --- | --- | --- |
| Metwally et al., 2016 | Y | Y | Y | unclear | N | N | Y | Y | N | N | unclear | 5 | Moderate |
| Kiros et al., 2025 | Y | Y | Y | unclear | N | Y | Y | Y | Y | Y | unclear | 8 | High |
| Park et al., 2014 | Y | Y | N | Y | N | N | Y | Y | N | Y | unclear | 6 | Moderate |
| Pan et al., 2025 | Y | Y | Y | Y | N | Y | Y | Y | Y | N | unclear | 8 | High |
| Huang et al., 2019 | Y | Y | N | unclear | N | N | Y | Y | N | N | unclear | 4 | Moderate |
| Reynolds et al., 2014 | Y | Y | N | Y | N | N | Y | Y | Y | N | unclear | 6 | Moderate |

**Notes:** ① Source of data clearly stated; ② Inclusion/exclusion criteria for exposure group provided; ③ Timing of participant identification clarified; ④ Continuity of study population if not population-based; ⑤ Assessment not biased by subjective factors; ⑥ Measures taken to ensure quality described; ⑦ Reason for exclusion of participants explained; ⑧ Confounding assessment/control described; ⑨ Handling of missing data explained; ⑩ Response rate and data completeness summarized; ⑪ Proportion of incomplete follow-up or outcomes for follow-up studies clarified. "Y" = Yes (1 point); "N" = No or Unclear (0 points). Total score: 0–3 = low, 4–7 = moderate, ≥8 = high quality.

Supplementary Figure S1. Sensitivity analysis for overall behavioral problems.

Supplementary Figure S2. Sensitivity analysis for internalizing behavioral problems.

Supplementary Figure S3. Sensitivity analysis for externalizing behavioral problems.

Supplementary Figure S4. Exploratory funnel plot for externalizing behavioral problems.
